# Total Abdominal Hysterectomy in a Young Woman With Refractory Abnormal Uterine Bleeding of Multifactorial Etiology: A Case Report of an Incidental Hemorrhagic Ovarian Cyst in a Single Ovary

**DOI:** 10.7759/cureus.106780

**Published:** 2026-04-10

**Authors:** Tahiana L de La Cruz, Krystel P Mateus, Adel J Tawil, Rommel F Portilla, Carlos Valladares

**Affiliations:** 1 General Medicine, Pontificia Universidad Católica Madre y Maestra (PUCMM), Santo Domingo, DOM; 2 General Medicine, Universidad Ricardo Palma (URP), Lima, PER; 3 General Medicine, Universidad de Panamá, Ciudad de Panamá, PAN; 4 Medicine, Universidad de las Américas (UDLA), Quito, ECU; 5 Pulmonary and Critical Care, University of Miami Miller School of Medicine, Miami, USA

**Keywords:** abdominal hysterectomy, abnormal uterine bleeding, case report, gynecologic surgery, histopathology, hypertension, leiomyomas, myomectomy alternatives, ovarian preservation, uterine fibroids

## Abstract

Uterine fibroids are the most common benign tumors of the female reproductive system and a frequent cause of abnormal uterine bleeding, pelvic pain, and reproductive morbidity in women of reproductive age. Their clinical management should be individualized according to symptoms, fibroid characteristics, comorbidities, and reproductive goals. A 33-year-old woman, gravida 5 para 4 with one prior abortion, with chronic hypertension controlled with candesartan and a history of multiple prior abdominal surgeries, including three cesarean sections and right oophorectomy, presented with persistent abnormal uterine bleeding refractory to medical therapy. Clinical and imaging evaluation revealed a solitary uterine fibroid and the absence of the right ovary. Endometrial biopsy showed secretory endometrium with focal irregular maturation and a minute fragment of squamous epithelium with koilocytic atypia, interpreted as likely cervical or vaginal contamination. After multidisciplinary assessment, total abdominal hysterectomy with wedge resection of a hemorrhagic cyst in the left ovary and uterosacral ligament suspension following the principles of McCall culdoplasty was performed. The procedure was completed without complications, with minimal blood loss. The postoperative course was uneventful, and the patient was discharged on the second postoperative day. Histopathological analysis confirmed intramural leiomyomas, benign hemorrhagic cyst, chronic nonspecific cervicitis, and irregular endometrial maturation without malignancy. This case illustrates the complexity of managing persistent abnormal uterine bleeding in a young woman with multiple prior abdominal surgeries, unilateral oophorectomy, and chronic hypertension. Although conservative options may be considered in selected patients, the combination of refractory symptoms, surgical history, and the need to preserve ovarian function supported a definitive surgical approach. Open abdominal hysterectomy allowed safe and controlled management, while partial preservation of the remaining ovary helped maintain endocrine function and reduce the risk of premature menopause. Total abdominal hysterectomy with partial ovarian preservation can be a safe and definitive option in young women with persistent abnormal uterine bleeding, uterine fibroids, prior abdominal surgeries, and associated risk factors. This case underscores the value of individualized surgical planning and multidisciplinary decision-making to achieve favorable outcomes.

## Introduction

Uterine fibroids, also known as leiomyomas, are the most common benign tumors of the female reproductive system and represent a frequent cause of abnormal uterine bleeding, pelvic pain, and reproductive problems in women of reproductive age [1,2]. Their prevalence varies across populations, with estimates indicating that 70-80% of women will develop fibroids during their lifetimes [1].

These tumors originate from the smooth muscle cells of the myometrium, and their growth is strongly influenced by hormonal factors, particularly estrogen and progesterone [3]. Beyond their high prevalence, leiomyomas impose a considerable public health burden due to their association with anemia, obstetric complications, and significant impairment in quality of life, including psychological complications [4]. Uterine fibroids can significantly affect reproductive outcomes, being associated with infertility, recurrent pregnancy loss, and complications during pregnancy and delivery, which underscores the need for individualized management strategies in women of reproductive age [5].

The International Federation of Gynecology and Obstetrics (FIGO) classification is a key tool for the evaluation and management of fibroids, as it provides a standardized description of their location and characteristics, which is essential for therapeutic planning [6]. Treatment options may include medical, surgical, or minimally invasive approaches, depending on symptoms, fibroid size and location, and the patient’s preferences and reproductive goals [1].

This case describes a 33-year-old woman with chronic abnormal uterine bleeding in the context of uterine fibroids and endometrial dysfunction, multiple prior abdominal surgeries, right oophorectomy, and long-standing hypertension. Relevant clinical features and therapeutic strategies are discussed, emphasizing the importance of comprehensive, patient-centered management.

## Case presentation

A 33-year-old woman, with four cesarean deliveries and one prior abortion, presented with persistent abnormal uterine bleeding for more than one year, refractory to medical treatment, in the context of documented uterine fibroids. Her medical history was notable for chronic hypertension since adolescence, well controlled with candesartan 16 mg daily. Her surgical history included three cesarean sections (2009, 2013, 2015), appendectomy (2019), right oophorectomy, and herniorrhaphy (2023).

On admission, she was alert and oriented, afebrile, with stable vital signs: blood pressure 120/80 mmHg, heart rate 85 bpm, respiratory rate 18 breaths per minute, and temperature 37°C. Physical examination revealed symmetrical, pendulous breasts without masses or lymphadenopathy. The abdomen was globose due to adipose panniculus, non-tender, and showed McBurney, periumbilical, and Pfannenstiel scars, with a body mass index calculated from anthropometric data of 36 kg/m², consistent with class II obesity. Cardiopulmonary examination was unremarkable. Pelvic support was preserved, and the lower extremities were symmetrical, mobile, and without edema. Speculum examination revealed intact vaginal and cervical mucosa without lesions. On bimanual examination, the cervix was posterior, 2 cm in length, with a closed internal OS; the uterus was anteverted and measured 7 cm; the adnexa showed no palpable masses.

**Figure 1:**
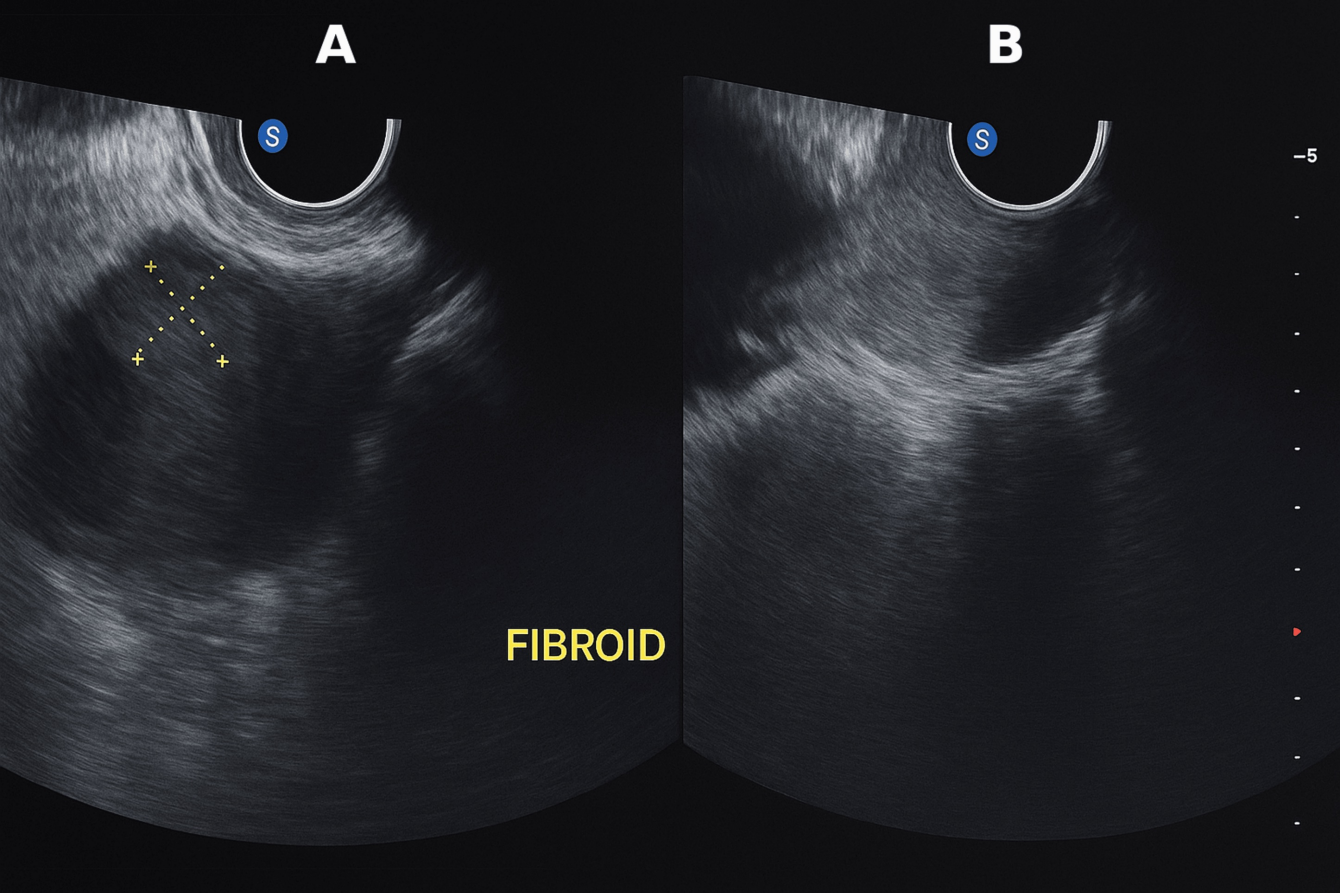
Transvaginal ultrasound, longitudinal plane (A) Intramural uterine fibroid measures 1.5 × 1.1 cm. It is outlined by yellow caliper crosshairs. The fibroid is hypoechoic, well-defined, and embedded within the myometrium. (B) The corresponding longitudinal image, shown without calipers, illustrates the fibroid's intramural position relative to the endometrium and serosal contour. The depth scale is visible on the right.

**Figure 2:**
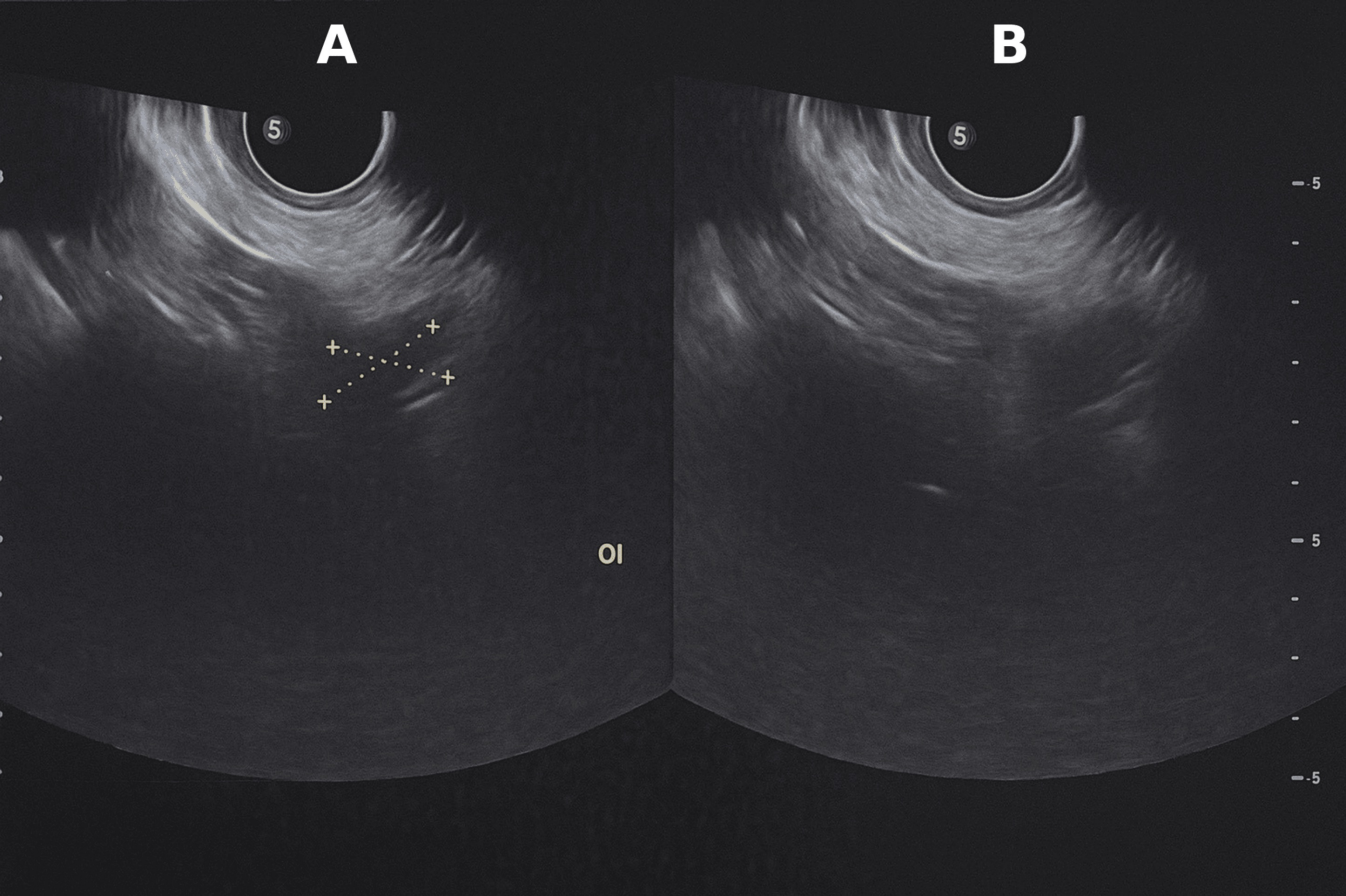
Transvaginal ultrasound, adnexal evaluation (A) Left ovary measured at 1.8 × 1.6 cm (yellow caliper marks), with preserved ovarian morphology. (B) Nonvisualization of the right ovary, consistent with prior right oophorectomy (depth scale at right margin).

Laboratory evaluation showed a complete blood count and metabolic profile within normal limits, except for mild anemia attributed to persistent abnormal uterine bleeding in a multifactorial clinical context [6,7]. Transvaginal ultrasound confirmed a single uterine fibroid (Figure 1) and the absence of the right ovary (Figure 2), consistent with a prior oophorectomy. The ovaries assessed on ultrasound were normal in size, with no evidence of cystic lesions on the preoperative study. A case has been reported of a patient with prior hysterectomy and unilateral oophorectomy who presented with pelvic pain and a pelvic mass that could not be conclusively characterized on imaging; only during laparoscopy was a hemorrhagic cyst identified and removed from the ovarian remnant, confirmed by histopathology [8]. A two-dimensional echocardiogram revealed normal cardiac function. Endometrial biopsy showed secretory-phase endometrium with focal irregularities and koilocytic atypia, the latter interpreted as probable contamination from cervical or vaginal epithelium during sample collection.

Due to persistent bleeding and the presence of irregular endometrial findings in the overall clinical context of refractory abnormal uterine bleeding, a total abdominal hysterectomy with wedge resection of a hemorrhagic cyst in the left ovary was scheduled. Preoperative evaluation by cardiology, anesthesiology, and internal medicine cleared the patient for surgery.

Although the fibroid was small, total hysterectomy was indicated due to persistent bleeding refractory to medical treatment, endometrial findings consistent with irregular maturation, history of multiple cesarean sections, prior oophorectomy, and chronic hypertension. This case emphasizes that although the FIGO PALM-COEIN classification guides the etiology of AUB, therapeutic decisions must be individualized, taking into account the overall clinical context [6].

In the preparatory phase, 1 g of cefazolin and 40 mg of omeprazole were administered, and antihypertensive treatment with candesartan was continued. Under subarachnoid block, a Pfannenstiel incision was chosen despite the history of multiple prior abdominal surgeries, following careful preoperative risk-benefit assessment. This choice was based on the fact that the pathology was confined to the pelvis, allowing adequate, safe, and controlled access through a low transverse suprapubic incision.

Additionally, the Pfannenstiel incision is associated with lower rates of surgical wound complications, reduced infection risk, lower incidence of incisional hernias, and improved functional recovery, even in patients with prior surgical history, when surgery is limited to the pelvic compartment.

Careful dissection along anatomical planes was performed, anticipating possible adhesions, which allowed adequate exposure of the surgical field. The uterus, measuring approximately 7 cm and containing an intramural fibroid, was removed. A hemorrhagic cyst was identified in the left ovary, and wedge resection was performed, a procedure that constitutes a form of partial oophorectomy, as it involves selective removal of the lesion while preserving surrounding functional ovarian tissue [9]. The absence of the right ovary, previously removed, was confirmed. A uterosacral ligament suspension following the principles of McCall culdoplasty was performed, in which the uterosacral ligaments are sutured to the vaginal cuff to reduce the risk of enterocele and vaginal vault prolapse after hysterectomy; this procedure can be adapted to abdominal hysterectomy as a prophylactic measure for apical prolapse, especially in patients with risk factors. The abdominal approach was chosen due to multiple prior surgeries and probable adhesions. Uterine vessels and supporting ligaments were ligated with Vicryl No. 1. Intraoperative blood loss was 200 mL, and no transfusion was required.

In the postoperative period, the patient remained hemodynamically stable and afebrile. Due to high infectious risk associated with class II obesity (BMI 36 kg/m²) and a history of multiple prior abdominal surgeries, perioperative antibiotic therapy was administered, including cefazolin as preincisional prophylaxis, followed postoperatively by ceftriaxone (1 g every 12 hours) and gentamicin (160 mg every 24 hours) as extended coverage in a complex, high-risk surgical setting. The therapeutic protocol was complemented by nalbuphine every six hours, acetaminophen as needed, ascorbic acid (500 mg IV every 12 hours), and thromboprophylaxis with enoxaparin (40 mg subcutaneously every 24 hours, initiated eight hours postoperatively). Management of her chronic condition with candesartan (16 mg/day) was continued.

On postoperative day 2, the patient showed favorable evolution, with stable vital signs and adequate pain control, and was discharged. Final histopathological analysis reported a cervix with chronic nonspecific cervicitis and an endometrial cavity containing fragments of endometrium with irregular maturation, explaining the abnormal uterine bleeding, along with a small fragment of squamous epithelium with koilocytic atypia, considered secondary to cervical or vaginal contamination. The ovary confirmed a benign hemorrhagic cyst, and the uterus showed intramural leiomyomas. These findings confirmed the benign nature of the process and validated surgery as the definitive solution for the patient’s condition.

## Discussion

This case highlights the complexity of managing symptomatic uterine fibroids in a young woman with multiple prior abdominal surgeries, unilateral oophorectomy, and chronic hypertension. Although uterine artery embolization and myomectomy remain valuable alternatives for fertility preservation, their application was limited in this patient due to her extensive surgical history, recurrent scarring, and irregular endometrial findings. In such cases, hysterectomy represents the most definitive and safest treatment option.

The FEMME trial (2022) demonstrated that both myomectomy and uterine artery embolization improve quality of life in women with fibroids, although myomectomy offers greater benefit at two years [10]. However, in our patient, repeated abdominal surgeries and endometrial pathology limited conservative options. Therefore, hysterectomy was the most appropriate and definitive treatment.

Although laparoscopic hysterectomy can reduce blood loss and recovery time even in patients with prior abdominal surgery [11], this case illustrates that patient-specific factors may outweigh general trends. In this context, an open abdominal approach provided greater surgical control and safety.

A case of successful myomectomy of a giant uterine leiomyoma has been reported, preserving the uterus despite the extreme tumor size [12]. Our patient, with three prior cesarean sections, multiple abdominal surgeries, and irregular endometrial findings, required a hysterectomy as the safest and most definitive method. This comparison highlights the importance of individualized surgical planning adapted to clinical complexity.

A key intraoperative challenge was the hemorrhagic cyst in the remaining ovary. In young women, preservation of ovarian parenchyma reduces the risk of premature menopause and its cardiovascular and metabolic consequences. In patients with prior contralateral oophorectomy, maintaining functional tissue is essential to preserve hormonal production. Thus, wedge resection balanced definitive treatment with endocrine preservation [9]. Recent evidence supports partial ovarian preservation in benign disease as a safe strategy with favorable long-term outcomes [13]. This underscores the importance of considering hormonal and metabolic consequences in young patients.

An extensive surgical history is associated with increased bleeding and complications during hysterectomy [14]. However, through multidisciplinary planning, regional anesthesia, antibiotic prophylaxis, anticoagulation, and multimodal analgesia, the patient recovered rapidly and was discharged on postoperative day 2 without complications.

Hypertension, present in this case since adolescence, has been reported to be associated with uterine fibroids in epidemiological studies [15]. However, the direction of this relationship remains complex and not fully established. Some evidence suggests that uterine fibroids may contribute to increased blood pressure through the secretion of growth factors and vasoactive peptides such as endothelin-1 [16]. In our patient, the coexistence of fibromatosis and chronic hypertension since adolescence reflects this multifactorial interaction.

The pathophysiology of uterine fibroids, including hormone-driven cellular proliferation, extracellular matrix accumulation, and abnormal angiogenesis, has been associated with persistent abnormal uterine bleeding [3,16]. In this case, fibroid size and location, along with histopathological endometrial findings, must be interpreted within the overall clinical context, given that technical factors during sample collection may influence certain microscopic findings, such as cervical or vaginal contamination.

Consequently, the indication for hysterectomy was based on persistent bleeding refractory to medical treatment and its clinical impact, rather than on an isolated histopathological finding.

In summary, this case highlights that in young women with uterine fibroids and complex surgical histories, individualized planning is essential. In our patient, hysterectomy with ovarian preservation through partial oophorectomy achieved definitive bleeding control without postoperative complications and avoided premature menopause. It should be noted that, given the benign nature of the hemorrhagic cyst, other options such as cystectomy or drainage with hemostasis could also have been considered. These findings suggest that even in complex surgical settings, hysterectomy can be safely performed when the approach is carefully selected and that partial ovarian preservation may be considered as a strategy to balance definitive treatment with long-term endocrine and metabolic health.

## Conclusions

This case illustrates the management of a young patient with persistent abnormal uterine bleeding refractory to medical treatment in the context of uterine fibroids, endometrial dysfunction, multiple prior abdominal surgeries, unilateral oophorectomy, and well-controlled hypertension. Total abdominal hysterectomy with wedge resection of a hemorrhagic cyst achieved definitive symptom control while preserving ovarian tissue and preventing premature menopause. The outcome highlights the importance of individualized surgical planning and multidisciplinary collaboration, with partial ovarian preservation representing a valuable option in young women with benign disease.
